# *Listeria* spp. in Street-Vended Ready-to-Eat Foods

**DOI:** 10.1155/2011/968031

**Published:** 2011-12-12

**Authors:** Moustafa El-Shenawy, Mohamed El-Shenawy, Jordi Mañes, Jose M. Soriano

**Affiliations:** ^1^Department of Food and Environmental Microbiology, National Research Center, Dokki, Cairo 12311, Egypt; ^2^Department of Environmental Microbiology, National Institute of Oceanography and Fisheries, Al-Anfushy, Alexandria 11695, Egypt; ^3^Observatory of Nutrition and Food Safety in Developing Countries, Department of Preventive Medicine and Public Health, Faculty of Pharmacy, University of Valencia, Burjassot 46100, Spain

## Abstract

Street-vended ready-to-eat food sold in Egypt, including sandwiches and dishes of traditional food, was examined for the presence of *Listeria* species. Out of 576 samples, 24% were found to contain *Listeria* species. *L. monocytogenes* and *L. innocua* were isolated from 57% and 39% of the contaminated samples, respectively. Other *Listeria* spp. were detected with lower frequency. *L. monocytogenes * of ≥10^3^ CFU/g were detected in 7% of the total examined samples, which represent 49% of the contaminated food samples (meat, poultry, seafood, dairy products, and products of plant origin). Most of the samples contaminated by *L. monocytogenes* had high levels of total viable bacterial counts. The results obtained may help to clarify the epidemiology of listeriosis in the country and draw the attention of the decision makers to issue hygienic regulations for food processing industries as well as street vendors in order to ensure safe street-vended ready-to-eat food.

## 1. Introduction

In Egypt, people eat foods that include sandwiches and variety of hot dishes which are mostly sold by street vendors. Street vendors, in Egypt, usually have small mobile carts with cabinets manufactured from wood, and sometimes covered with glass, to protect their foods from the environmental contamination. They always use utensils manufactured from aluminum, stainless steel, and plastic to prepare and serve their foods. Vendors sell sandwiches prepared from ready-to-eat fillings such as luncheon (processed, repacked meat/chicken often molded into a loaf and served sliced), basturma (air-dried seasoned beef), salmon, tuna, hard cheese (Romy), white soft cheese (Damietta), processed cheese, and cream-based sandwiches in addition to ice cream. Other fillings are prepared in streets by boiling or frying meat products, seafood, poultry, eggs, fuol medames (overnight boiled faba beans), falafel (fried paste of faba beans, green vegetables, onion, garlic, and other spices) as well as koshary dishes, which consist of rice, macaroni, black lentil, and fried onion.

The hot climate and the environmental conditions such as the dusty streets, in which these vendors are working, favor bacterial growth, and there may be uncertainty about which food item is finally served to the consumer. Furthermore, the lack of refrigeration facilities, sources of running water, personal hygiene, and public toilets may increase the chances for cross-contamination.

The cross-contamination of such foods with pathogenic microorganisms could occur during the processing of ready fillings as well as during the preparation of fillings and sandwiches, in vendor's homes and cars [1–6].

Until now, only one study, in Egypt, on the assessment of foodborne pathogens in street-vended ready-to-eat foods has been done [7]. El-Sherbeeny and his colleagues [7] examined 114 samples, which were collected from the mobile carts over a period of 3 years, to find bacterial contamination; their study revealed that 41%, 37%, 26%, and 3% of the samples were contaminated with *Staphylococcus aureus*, *Bacillus cereus*, *Clostridum perfringens*, and *Shigella*, respectively. Notably, in none of the samples, *Vibrio parahaemolyticus* and *Salmonella*—two well-known foodborne pathogens—were detected. However, the presence of *Listeria* spp. has never been investigated in these kinds of food.

The bacterium *Listeria* is widely distributed in our environment. Its most important species is *Listeria monocytogenes* that has been long recognized as the cause of a serious human disease, known as listeriosis [8]. Clinical manifestations of this disease include abortion, meningoencephalitis, and septicaemia in immunocompromised individuals [9–11]. Listeriosis can be resulting from the consumption of *L. monocytogens* contaminated foods such as meat, poultry, dairy products, seafood, and other types of food. Street-vended foods can be easily contaminated with this pathogen as the organism is widespread in nature and can overlap the environmental conditions [12–18]. The infectious dose of *L. monocytogens* can vary depending on several factors, of which the immune status of the individual and the virulence of the strain ingested are among the most important [11]. Currently, there is a lack of information on the occurrence of foodborne diseases in Egypt. The reported cases done by the Department of Infectious Diseases in the Ministry of Health do not reflect the actual occurrence of foodborne diseases in Egypt, since notification is required only when five or more cases are reported by either one physician or one medical institution (personal communication). Thus, the foodborne transmission of infectious diseases and their causative agents remain unknown in most cases.

 In view of the open and unhygienic environmental conditions, in which street-vended foods are prepared and handled, contamination of these foods with *Listeria* spp., including *L. monocytogenes*, seems likely. However, the prevalence of *L. monocytogenes* and the epidemiology of the other *Listeria* spp. in these foods are not known. Thus, work was undertaken to assess the extent to which ready-to-eat foods sold by street vendors could expose consumers to *L. monocytogenes *and other* Listeria *spp.

## 2. Material and Methods

### 2.1. Sampling

The prepared sandwiches and dishes were purchased from the mobile carts in the city of Alexandria. Additionally, other types of food such as shawarma (similar to kebab), chicken, shrimp and wrapped ice cream were purchased from the fixed carts, which were associated with a variety of small shops and restaurants. All samples were aseptically transferred into plastic bags, and the bags were maintained inside ice boxes (6–8°C) during transportation to the laboratory. The plastic bags containing food samples were classified into five groups, according to their major components other than bread (Table 1). All samples were processed within 4 h of collection.

### 2.2. Microbiological Analysis

Constituents of each food sample were mixed well, using sterile porcelain horn manually, in their plastic bag. Then, 25 g of each sample was aseptically weighed using sterile spatula and blended for 1 min with 225 mL of buffered peptone water in a sterile stainless steel blender cap [14]. The homogenate was transferred to a sterile bottle jar and was incubated at 30°C for 24 h. Ten mL of the incubated homogenate was added to 90 mL of *Listeria *Selective Broth (Oxoid) and further incubated for 48 h at 30°C. Thereafter, the homogenate was streaked onto two plates of Oxford agar (Oxoid), incubated for 48 h at 37°C. Grey colonies surrounded by black zones were presumed to be *Listeria*.

For determination of *Listeria *counts using the direct method [10], the original homogenates were decimally diluted using peptone water, and 0.1 mL of the appropriate dilutions were spread onto duplicate plates of Oxford agar and incubated at 37°C for 48 h. Grey colonies surrounded by black zones were counted. For determination of total viable counts (TVCs), 1 mL portions of suitable dilution of the original homogenates were pouring plated in duplicate using total plate count agar (Difco). Plates were incubated at 30°C for 72 h. All bacterial counts were recorded as CFU/g.

### 2.3. Identification of Isolates

At most, five presumptive *Listeria* colonies were picked from each selective agar plate. These colonies were purified using Trypticase soy agar (Difco) and submitted to the Henry illumination test [19]. A pure culture of *L. monocytogenes* V7 strain serotype 1 (milk isolate), obtained from the Department of Food Science, University of Wisconsin, Madison, USA served as a control. Nonspore forming Gram-positive coccobacilli (single and/or in chains) isolates were tested for catalase and umbrella growth in Motility test medium (Difco) at 25°C. Isolates positive for both the tests were further examined for hemolysin production using Tryptose agar (Oxoid) with 5% sheep blood (obtained from Faculty of Veterinary Medicine, Cairo University). The isolates were streaked onto Tryptose Agar (TA) slants, incubated at 35°C for 24 h, and then they were identified at the species level using the criteria of McLauchlin [2] as described by Bell [20]. The isolates were tested for fermentation of rhamnose, xylose and mannitol, CAMP test (synergistic lysis of red blood cells) against *S. aureus* ATCC 25923, and API *Listeria*. Moreover, the isolates from TA plates were confirmed by slide agglutination using polyvalent *Listeria *O antiserum (Difco).

### 2.4. Statistical Analysis

The results were analyzed by means of the chi-square analysis (SAS, [21]) to determine the relationship between the type of the food groups and the presence of either *Listeria* spp. or *L. monocytogenes*.

## 3. Results and Discussion

In this study, a total of 576 samples (24 samples from each of the 24 food items belong to five major food groups) were examined. The results were summarized in Table 1. *Listeria *spp. were detected in all the five food groups. They were recovered from 24% (137 samples) of the total examined samples. Using the direct count technique, seven samples were found to be negative for the presence of *Listeria* spp., but were found positive after enrichment. Out of the 137 *Listeria *spp. contaminated samples, 78 samples were found to be contaminated with *L. monocytogenes* and 54 samples harboured *L. innocua*, however *L. seeligeri *and* L. welshimeri *were detected in two samples each. The *Listeria* species isolated from the remained one food sample could not be identified. Among the 78 *L. monocytogenes* contaminated samples, 22, 18, 14, 9, 7, and 8 samples were found to harbor the pathogen in levels of <200, 200–<10^3^, ≥10^3^-10^4^, >10^4^-10^5^, >10^5^-10^6^, and >10^6^ CFU/g, respectively. The levels of the total viable counts in relation to levels of contamination by *L. monocytogenes* are illustrated in Table 2.

The statistical analysis revealed that there is a relationship between the type of food groups and the presence of either *Listeria* spp. or *L. monocytogenes* (*P* < 0.05). In the meat products group, isolation of *L. monocytogenes* was higher in sandwiches of minced meat (25%), sausage (21%), and shawarma (21%) than the other type of sandwiches (Table 1). In this group, there was a relationship between the type of sandwiches and the isolation rate of the pathogens (*P* < 0.05). The same was true with the poultry products, where chicken (17%), luncheon (17%), and shawarma (12%) recorded the highest contamination (*P* < 0.05). In the seafood products group, higher prevalence of *L. monocytogenes* was detected in sandwiches of shrimp (21%) and Sepia (21%). In dairy products group, there was no relationship between the type of sandwiches and the presence of *L. monocytogenes* (*P* > 0.05), although the pathogen was isolated from 25%, 25%, and 21% of sandwiches of hard cheese, cream with honey, and white soft cheese, respectively. In plant products group, there was a relationship between the type of the examined food products and the presence of the pathogen (*P* < 0.05), in which higher prevalence of *L. monocytogenes *was detected in koshary dishes (38%) and falafel (12%). In fact, there are many combined conditions and factors that affect the presence/survival and growth of the bacterium in the food products [20]; therefore, it is difficult to explain the observed relationship between the food groups and the food type/items.

Overall, the results obtained, in this study, are similar to the findings by other authors [6, 11, 22–26]. Depending on the food group, 8 to 24% of the investigated samples were found to harbor the pathogen (Table 1). Our findings are consistent with the results obtained by Cabedo et al. [26], who found *L. monocytogenes* in ready-to-eat foods at a range of 6.2% to 20.0%, depending on the food products.

Notably, seven samples were found negative for the presence of *Listeria* spp. by direct count technique. However, the same samples appeared as positive for the bacterium by following an enrichment procedure prior to the direct count technique. In this regard, Wilson [13] mentioned that it is likely that poor cooling temperature is the major factor in the continued occurrence of *Listeria *spp. in products when the organisms are detected by direct count. Thus, in our results, the fact that the most *Listeria*-positive samples (130 out of 137) detected by direct plating might be indicative of temperature abuse, as may be expect from street vending facilities in Egypt with no or limited refrigeration capabilities.

Our results indicated that *L. monocytogenes* and *L. innocua* were the most commonly recovered species, although other species were also detected, but in lower frequency. In the United Kingdom, *L. monocytogenes *was found to be more common in variety of ready-to-eat foods than other kinds of food [12]. However, in another study, both *L. monocytogenes* and* L. innocua* were the only species isolated in prepared retail sandwiches [13]. In the same country, a comprehensive survey of ready-to-eat foods by Wilson [27] found that most of the *Listeria *spp. isolated was *L. monocytogene*s (49%) and *L. innocua* (36%) and other species were found at lower levels. By contrast, a high prevalence of *Listeria* spp. other than *L. monocytogenes* and *L*. *innocua* was detected in ready-to-eat foods at the Belgian market [28]. Although our results are similar to those of the United Kingdom, the differences in the data related to occurrence and distribution of the genus *Listeria* might be explained by the geographical/environmental specificity [29]. *L. monocytogenes* was also found to be more prevalent than *L. innocua *in food products in warm/hot countries, such as Brazil [30], India [31], and Mexico [32].

Concerning the permissible limits of *L. monocytogenes *in ready-to-eat foods, in the United Kingdom and the European Union, the upper limit now is <100 CFU/g (testing at the end of shelf life) in products at the market [33]. However, the United States adopted a zero tolerance for the presence of *L. monocytogenes* in ready-to-eat products [34]. More older, the British PHLS criteria [35] suggested that food containing 10^2^ to 10^3^ CFU/g, which indicates a nonsignificant failure of hygienic standards in the preparation and/or storage of such foods, should be considered unsatisfactory. However, those contaminated with ≥10^3^ CFU/g, which indicate a significant failure of hygienic standards in the preparation and/or storage of such foods, should be considered potentially hazardous. According to these provisional guidelines, in this study, potentially hazardous level of *L. monocytogenes* (≥10^3^ CFU/g) was found in 38 sandwiches/dishes, which represent about 7% of the total samples and 49% of the samples contaminated by *Listeria *spp. Additionally, this hazardous level of *L. monocytogenes* was also found in samples from all the food groups (Table 1). Such sandwiches/dishes represent a significant risk of exposure to consumers, especially koshary dishes, which are popularly consumed.

Detectable levels of *L. monocytogenes* (10^2^-10^3^) were found in 18 food products which represented about 3% of the total examined samples and 23% of the contaminated samples. At the same time, 4% of the total samples and 28% of the contaminated samples had *L. monocytogenes* counts less than 200 CFU/g.

An association between the presence of *L. monocytogenes *and high TVCs (>10^4^) is evident from Table 2. The data show that only 6 samples, representing 5% (6 out 120 samples) of the total samples that had TVCs of <10^4^, were found to be contaminated with *L. monocytogenes.* However, in samples that had TVCs > 10^4^ CFU/g, this percentage has increased to 16% (72 out of 456 samples). The same was true between high TVCs and the presence of higher levels of *L. monocytogenes *(potentially hazardous) since 35 out of 38 samples which had ≥10^3^ CFU/g *L. monocytogenes *had also TVCs > 10^4^ CFU/g (Table 2). Although the present work clearly indicated that there was an association between high TVCs and the presence of the pathogen, it is unsafe to assume that there are no exceptions to this trend, since 6 sandwiches having TVCs < 10^4^ CFU/g harbored the pathogen (Table 3). Such association may be due to poor hygiene during filling manufacturing, postprocess environmental contamination after cooking, and/or poor personal hygiene of the vendors themselves. Moreover, bread [27] and some other food ingredients commonly used in the preparation of sandwiches such as butter and margarine [13] may be also a source of contamination.

The inefficient handling of already manufactured fillings and/or fillings prepared by the vendors themselves in the streets may represent an important source of contamination, since sandwiches made from canned fillings such as salmon and tuna were free from the pathogen. Food items, such as processed cheese, foul medames, fried eggs, and ice cream, which were exposed to high temperatures during cooking/processing, had a lower positive rate (Table 1).

Another serious reason of cross-contamination is that street vendors usually wash the utensils and dishes, used for preparation and serving their food, in buckets containing stagnant water which remains unchanged for the whole selling period and also exposed to the dust in the streets. Another important factor is that those vendors do not have facilities to keep fillings/foods at controlled low temperature. They store the unsold at room temperature at their home and sell those foods in the next day (personal communication with some vendors); such acts certainly favor bacterial growth in either raw or cooked fillings.

In conclusion, this work gives an indication about the presence of *Listeria* spp. in ready-to-eat foods sold in streets. The levels of contamination should be interpreted with caution since there are many factors that may affect these findings. These factors include the quantity of filling(s) found in each sandwich, the filling(s)/bread ratio, sandwich(s)/koshary dishes ratio, and the environmental condition of the streets where the food products were vended. Our results showed that the consumption of such contaminated street-vended ready-to-eat foods pose a serious to the public health. Results from this study will certainly help to clarify the epidemiology of foodborne diseases in Egypt so that proper methods/actions can be taken to prevent and control outbreaks. It may also be useful in the development of microbiological guidelines. We believe that hygienic regulations should be issued for these street vendors, since most of them are not inspected by the concern authorities. On the other hand, careful handling/preparing, washing, cleaning of foods, and above all personal hygiene awareness would help to minimize or prevent contamination by such bacteria.

## Figures and Tables

**Table 1:** *Listeria *spp. detected in street-vended ready-to-eat foods by plate count method.

Food group(s)	Food item(s)^a^	No. of samples containing *listeria *spp. (%)	No. of samples containing *L. monocytogens* (%)	Prevalence of *L. monocytogens* in *Listeria* spp. (expressed in %)
Meat products	Shawarma	^ b(1)^8 (33)	^<4>^5 (21)	63
	Luncheon	^(1)^6 (25)	3 (12)	50
	Basturma	^(1)^3 (13)	2 (8)	66
	Liver	4 (17)	2 (8)	50
	Sausage	9 (38)	^<4>^5 (21)	56
	Minced meat	10 (42)	^<5>^6 (25)	60
Subtotal		40 (28)	23 (16)	58

Poultry products	Chicken	7 (29)	^<4>^4 (17)	57
	Luncheon	6 (25)	4 (17)	66
	Shawarma	5 (21)	3 (12)	60
	Boiled eggs	2 (8)	0 (0)	0
	Fried eggs	3 (13)	0 (0)	0
Subtotal		23 (19)	11 (9)	48

Seafood products	Shrimp	^(1)^8 (33)	^<5>^5 (21)	63
	Fried fish	4 (17)	0 (0)	0
	Sepia	7 (29)	5 (21)	71
	Salmon	0 (0)	0 (0)	0
	Tuna	0 (0)	0 (0)	0
Subtotal		19 (16)	10 (8)	53

Dairy products	Hard cheese	^(1)^9 (38)	^<3>^6 (25)	67
	White soft cheese	7 (29)	^<1>^5 (21)	71%
	Processed cheese	0 (0)	0 (0)	0
	Cream and Honey	9 (38)	6 (25)	67%
	Ice cream	2 (8)	0 (0)	0
Subtotal		27 (23)	17 (14)	63

Plant products	Ful Medames	3 (13)	0 (0)	0
	Falafel	5 (21)	3 (12)	60
	Koshary	^(2)^20 (83)	^<12>^14 (58)	70
Subtotal		28 (39)	17 (24)	61

Grand total		137 (24)	78 (14)	38

^a^Total 24 samples from each food item were examined.

^b( )^Number of samples positive for the presence of *Listeria *spp. only after enrichment.

^< >^Number of samples containing *L. monocytogens* of ≥10^3^ CFU/g.

**Table 2:** TVCs in relation to levels of contamination by *L. monocytogens*.

Levels of the total viable counts/gm	<10^4^	>10^4^-10^5^	>10^5^-10^6^	>10^6^
Total examined samples (**576** food samples).	120	192	144	120
Total samples containing *L. monocytogens *(**78** food samples)	6	19	24	29
Samples containing levels < 10^3^ CFU/g *L. monocytogens* (**40** food samples).	3	10	12	15
Samples containing levels ≥ 10^3^ CFU/g *L. monocytogens* (**38** food samples).	3	9	12	14

*TVC: Total Viable Count.
